# Electrochemical Determination of Caffeine Content in Ethiopian Coffee Samples Using Lignin Modified Glassy Carbon Electrode

**DOI:** 10.1155/2017/3979068

**Published:** 2017-04-23

**Authors:** Meareg Amare, Senait Aklog

**Affiliations:** Bahir Dar University, P.O. Box 79, Bahir Dar, Ethiopia

## Abstract

Lignin film was deposited at the surface of glassy carbon electrode potentiostatically. In contrast to the unmodified glassy carbon electrode, an oxidative peak with an improved current and overpotential for caffeine at modified electrode showed catalytic activity of the modifier towards oxidation of caffeine. Linear dependence of peak current on caffeine concentration in the range 6 × 10^−6^ to 100 × 10^−6^ mol L^−1^ with determination coefficient and method detection limit (LoD = 3 s/slope) of 0.99925 and 8.37 × 10^−7^ mol L^−1^, respectively, supplemented by recovery results of 93.79–102.17% validated the developed method. An attempt was made to determine the caffeine content of aqueous coffee extracts of Ethiopian coffees grown in four coffee cultivating localities (Wonbera, Wolega, Finoteselam, and Zegie) and hence to evaluate the correlation between users preference and caffeine content. In agreement with reported works, caffeine contents (w/w%) of 0.164 in Wonbera coffee; 0.134 in Wolega coffee; 0.097 in Finoteselam coffee; and 0.089 in Zegie coffee were detected confirming the applicability of the developed method for determination of caffeine in a complex matrix environment. The result indicated that users' highest preference for Wonbera and least preference for Zegie cultivated coffees are in agreement with the caffeine content.

## 1. Introduction

Alkaloids are broad category of nitrogen containing organic metabolites produced by plants; the plants that produce these alkaloids make their leaves unattractive to eating by insects and higher animals [1]. The awfully known toxic compounds morphine, quinine, cocaine, and codeine belong to this group of compounds. Caffeine (3,7-dihydro-1,3,7-trimethyl-1*H*-purine-2,6-dione) is one of the naturally occurring alkaloids that is widely contained in plant products and beverages. Caffeine is a natural stimulant contained in many sources like coffee, tea, chocolate, soft drinks, and tablets for the treatment of many diseases such as asthma, nasal congestion, and headache and even for improving athletic endurance and facilitating weight loss [1]. Many of caffeine consumers get caffeine from multiple sources, the caffeine content of which varies with the type of source and its amount [2–5].

However, reports on human and animal studies showed that caffeine produces mental and behavioral effects that are similar to those of typical psychomotor stimulant drugs such as amphetamine and cocaine [6]. Stimulation of the central nervous system, diuresis, gastric acid secretion, and increased blood pressure are among the reported physiological effects associated with caffeine [7]. Among the different possible sources, coffee is known to have the highest caffeine concentration and yet the most utilized source of caffeine [5].

The word coffee is believed to be derived from* Keffa*, which is the name of the locality in Abyssinia/Ethiopia where coffee beans were first discovered by Sheferds in the 6th century [8]. Since then, coffee has become one of the most widely consumed beverages throughout the world [9].

Coffee beans, which are seeds of Rubiaceae botanic family and* Coffea* genus [10], have two common species:* Coffea arabica *and* Coffea canephora* [11, 12]. While most of the coffee plants cultivated in Ethiopia are* Coffee arabica*, there are, however, wide ranges of variabilities including decaffeinated varieties in the country [2, 13, 14] which might be attributed to variation in the soil, altitude, and climate of the localities where they are grown [4, 15].

Among the coffee varieties in the country, coffees cultivated at four localities of Ethiopia, Finoteselam, Wolega, Zegie, and Wonbera, are commonly consumed by the consumers in Bahir Dar City, North West Ethiopia. Of these, the coffee cultivated in Wonbera locality is the most expensive and preferred while the Zegie coffee is the cheapest and least preferred, the basis for which might be psychological, aroma, otherwise the caffeine content. Thus, the aim of this research was to check whether the users' preference can be related to the caffeine content or not which needs determination of the caffeine content of the four coffee varieties using a sensitive, selective, fast, and environmentally friendly method.

High performance liquid chromatography [16, 17], capillary chromatography [18, 19], spectroscopy [20, 21], and electrochemical [22, 23] methods are among the reported methods for determination of caffeine in coffee, tea, and cola beverage samples. Most of the reported methods require carcinogenic organic solvents for extraction and hence are environmentally nonfriendly. Moreover, using time-consuming sample preparation procedures such as liquid-liquid extraction and solid-phase extraction or the use of more than one chromatographic step, costly instrumentation, and high skilled technician [12, 23] makes them inconvenient.

In contrast to the conventional analytical methods, electrochemical methods are powerful and versatile analytical techniques that offer high sensitivity, accuracy and precision, relatively wider linear dynamic range and low-cost instrumentation, specificity for a particular oxidation state of an element, relative inexpensiveness, and being usually environmentally friendly (use no or minimum amount of organic solvent) [23, 24].

In recent years, working electrodes including boron doped diamond [25–28], carbon paste and carbon nanotubes [29, 30], and glassy carbon [22, 31, 32] based electrodes have been reported for determination of caffeine in real samples.

Carbon materials have broad potential window, low background current, rich surface chemistry, comparative chemical inertness, relatively easy electrode preparation, and low cost, making them electrodes of choice [33]. Deliberate and controlled modification of the electrode surface can produce electrodes with new and interesting properties that may form the basis of new applications of electrochemistry [34]. Polymer-modified electrodes (PMEs) have received considerable attention in recent years due to their good stability, reproducibility, increased active sites, homogeneity in electrochemical deposition, and strong adherence to the electrode surface [35, 36].

Lignin is a natural polymer contained in most woody trees and shrubs [37]. Approximately 2% of the lignin released during chemical pulping in pulp and paper industries is used for chemical and material applications and the remainder is burned as boiler fuel showing its availability. Although the electrochemical behavior of lignin at the surface of glassy carbon electrode has been reported [38], to the best of our knowledge, its application for electrochemical determination of caffeine in aqueous extracts of coffee samples has not been reported. This paper reports a simple, cheap, and environmentally friendly lignin based electrochemical method for determination of caffeine in aqueous extracts of coffee samples cultivated in different localities of Ethiopia, the origin of coffee.

## 2. Experimental

### 2.1. Chemicals and Instruments

Caffeine (Fischer Scientific), H_3_PO_4_ (Cipla Ltd., India), K_2_HPO_4_ (Wagtech International Ltd., UK), CH_3_COOH and NaOH (Landmark chemicals PVT., India), HCl (Riedel-De Haen, Germany), lignin (Riedel-De Haen, Germany), HNO_3_ (65–70%), and H_2_SO_4_ (99%) (both from Loba chemie Pvt., Ltd.) were used. All reagents were of analytical grade and were used directly without further purification. Glassware was cleaned using chromic solution prepared by dissolving 1 g of potassium dichromate in 1 L of H_2_SO_4_ followed by rinsing with distilled water. Distilled water was used for the preparation of all solutions.

Voltammetric experiments were carried out using CHI760D Electrochemical Workstation (Austin, Texas, USA) connected to a personal computer. All electrochemical experiments were performed, employing a conventional three-electrode system with a glassy carbon electrode (3 mm in diameter) or a Lignin modified glassy carbon electrode as the working electrode, platinum coil as an auxiliary electrode, and Ag/AgCl as a reference electrode. All experiments were carried out at 20 ± 2°C.

### 2.2. Real Sample Preparation

Coffee samples were purchased from the respective local markets (Wolega, Finoteselam, Zegie, and Wonbera). Aqueous extracts of the coffee samples were prepared following the procedure outlined elsewhere [22]. Briefly, exactly 50 g of coffee from each sample was roasted by using conventional coffee roasting machine. Each roasted coffee sample was ground using a mortar and pestle and screened through 250 *μ*M sieve to get a uniform texture. An accurately weighed 4 g of sieved coffee from each sample was then boiled in 200 mL of distilled water in the temperature range 80–90°C for 30 min while stirring using magnetic stirrer. The water extracted caffeine was finally separated from the residue by decantation and was ready for the DPV analysis.

### 2.3. Preparation of Lignin Modified Glassy Carbon Electrode

The lignin modified glassy carbon electrode (LGCE) was prepared following a procedure reported elsewhere [38]. Briefly, a glassy carbon electrode (3 mm diameter) was first rinsed with distilled water and polished carefully with alumina powder, having different particle size (1.0, 0.3, and 0.05 *μ*m), to a mirror finish surface. The residual polishing material was removed by repetitive rinsing of the surface with distilled water. The resulting electrode was immersed in pH 7 Phosphate buffer solution (PBS) and was activated potentiodynamically by scanning the potential between −0.2 and +1.5 V for five cycles at a scan rate of 100 mV s^−1^. Then, the activated electrode was transferred to a cell containing lignin in a mixture of 0.5 M H_2_SO_4_ and 0.1 M HNO_3_ prepared by dissolving 10 mg of lignin in 50 mL of the mixture (1 : 1 volume ratio).

Lignin polymer was then deposited at the electrode surface at a potential of +0.9 V for 2 minutes. The lignin modified electrode was then rinsed with distilled water to remove physically adsorbed and unreacted species from the electrode surface. Subsequently, the modified electrode was stabilized in pH 7 PBS by scanning the potential between −0.2 V and +1.0 V until a steady cyclic voltammogram was obtained. Finally, the modified electrode was dried in air and made ready for use.

### 2.4. Preparation of Standard Solutions

For cyclic voltammetric studies, 10 mM stock solution of caffeine was prepared by dissolving 0.1941 g of caffeine in 100 mL of 0.1 M pH 5 Acetate buffer solution (ABS) [22]. From the stock solution, while 1 mM solution of caffeine was used for the voltammetric investigation of caffeine at both the unmodified and modified electrodes, working solutions of different concentrations of caffeine (6, 8, 10, 20, 30, 50, 60, 80, and 100 *μ*M) in pH 5 ABS were prepared from 500 *μ*M intermediate solution through serial dilution.

## 3. Results and Discussion

Cyclic voltammetric and differential pulse voltammetric techniques were used to investigate the electrochemical behavior of caffeine and determine caffeine content in coffee samples, respectively.

### 3.1. Cyclic Voltammetric Behavior of Caffeine at LGCE

**Figure 1:**
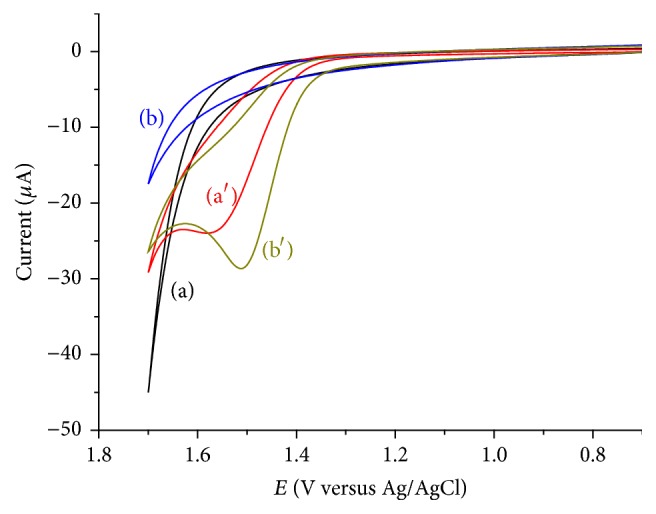
CVs of unmodified GCE (a and a′) and LGCE (b and b′) in pH 5 ABS containing no (a and b) and 1 mM (a′ and b′) caffeine.

Figure 1 presents the cyclic voltammograms of unmodified and modified glassy carbon electrodes in the absence and presence of caffeine.

As can be seen from the figure, no peak was observed at both the unmodified and modified glassy carbon electrodes in caffeine-free buffer solution. On the contrary, well resolved oxidative peak was recorded at the unmodified GCE and LGCE at a potential of 1.578 and 1.510 V, respectively. Absence of a reductive peak indicated that caffeine undergoes an irreversible oxidation at both the modified and unmodified electrodes which is in agreement with previously reported works [22, 36].

Appearance of an oxidative peak with a relatively larger peak current and improved peak potential at the lignin modified electrode compared with the unmodified GCE also showed the catalytic activity of the lignin modified electrode which might be due to an increased electrode surface area and/or improved electron exchange at the electrode surface [22, 38], making it suitable for caffeine analysis.

### 3.2. Effect of Scan Rate

**Figure 2:**
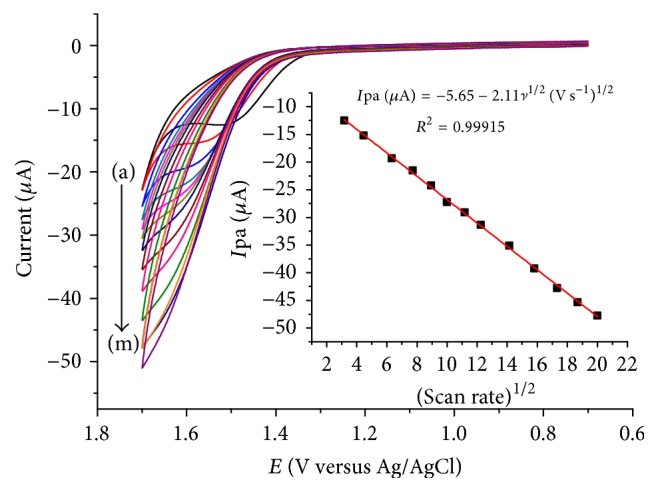
Cyclic voltammograms of lignin modified GCE at pH 5 ABS containing 1 mM caffeine at different scan rates ((a)–(m): 10, 20, 40, 60, 80, 100, 125, 150, 200, 250, 300, 350, and 400 mV s^−1^, resp.). Inset: plot of oxidative peak current as a function of the square root of scan rate.

To investigate the type of reaction kinetics the caffeine follows at the surface of lignin modified glassy carbon electrode, the determination coefficients (*R*^2^) for the plots of peak current as a function of scan rate and square root of scan rate were compared in the scan rate range 10–400 mV s^−1^ (Figure 2). As can be seen from the figure, the oxidative peak current shifted to a higher positive value with increasing scan rate, confirming the irreversibility of the oxidation reaction of caffeine at the modified electrode. Furthermore, a higher determination coefficient (*R*^2^ = 0.999) for the dependence of peak current on the square root of scan rate (inset of Figure 2) than for the dependence of peak current on the scan rate indicated that the oxidation of caffeine at the modified electrode follows diffusion controlled kinetics which is in agreement with reported works [33].

### 3.3. Effect of Solution pH on the Peak Current

**Figure 3:**
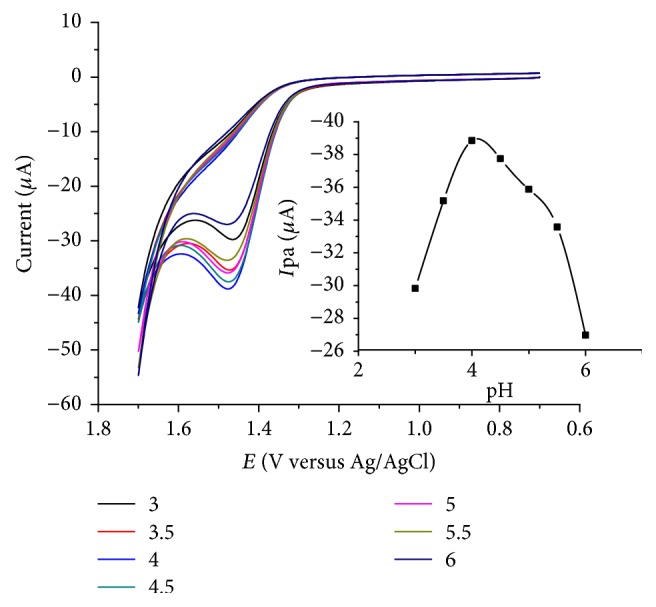
CVs of LGCE in ABS of different pH values (3.0–6.0) containing 1.0 × 10^−3^ mol L^−1^ of caffeine. Inset: plot of Ipa versus pH in pH range 3.0–6.0 at a scan rate of 100 mV s^−1^.

The effect of pH on the electrochemical response of lignin modified electrode for caffeine was studied in the pH range of 3 to 6.  Figure 3 presents the cyclic voltammograms of 1 mM caffeine in 0.1 M ABS of various pHs at LGCE. As can be observed from the figure, the oxidative peak current increased with pH 3 to 4 which then decreased in pH values beyond 4. Thus, pH 4 was selected as the optimum pH which was used in the subsequent experiments.

### 3.4. Differential Pulse Voltammetric Studies of Caffeine at LGCE

**Figure 4:**
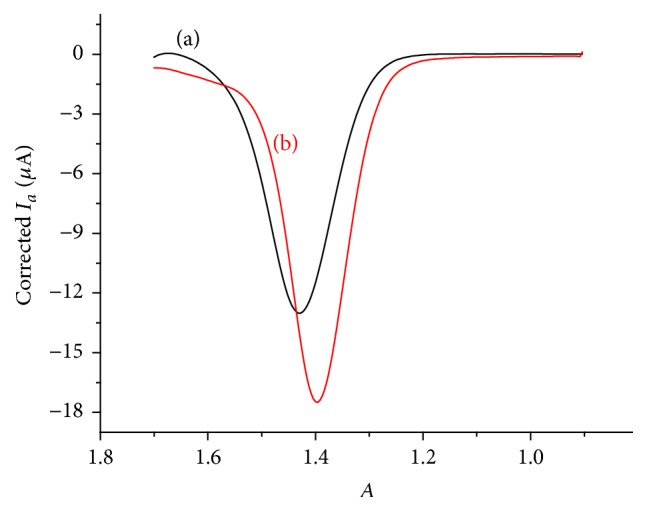
Differential pulse voltammograms (corrected for background) of unmodified (a) and modified (b) GCE in pH 4.0 ABS containing 1 mM caffeine.

Since it is an effective voltammetric method with established advantages, including good discrimination against background current and low detection limit, differential pulse voltammetry (DPV) was used for the quantification of caffeine content of coffee samples cultivated in different localities of Ethiopia.  Figure 4 presents differential pulse voltammograms of unmodified and lignin modified glassy carbon electrodes in pH 4 ABS containing 1 mM caffeine. An oxidative peak with an improved peak current and peak potential at the modified electrode (*curve* (b) of Figure 4) relative to at the unmodified electrode (*curve* (a) of Figure 4) confirmed the catalytic effect of the lignin modified glassy carbon electrode towards oxidation of caffeine.

### 3.5. Optimization of Method Parameters

**Figure 5:**
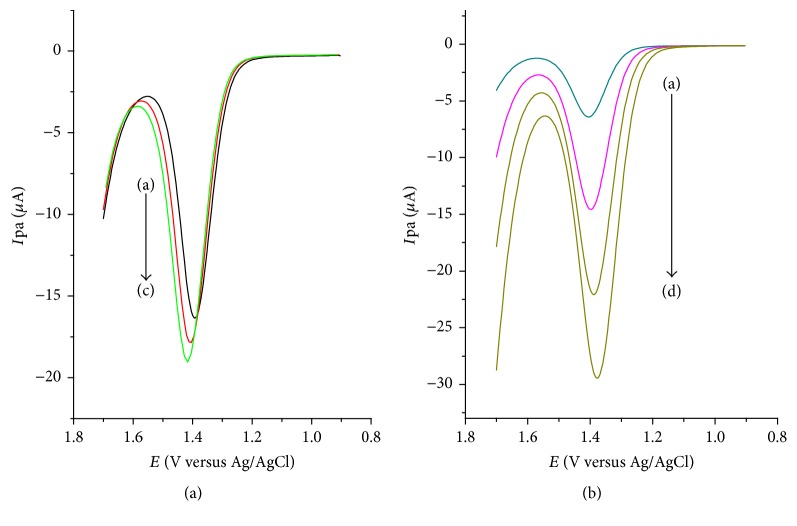
DPVs of lignin modified GCE in pH 4 ABS containing 1 mM caffeine at (a) different step potentials ((a)–(c): 4, 8, and 12 mV, resp.) and pulse amplitude of 50 mV and (b) different amplitudes ((a)–(d): 25, 50, 75, and 100 mV, resp.) and step potential of 8 mV.

The effect of differential pulse voltammetric parameters such as the step potential and pulse amplitude on the peak current response of lignin modified glassy carbon electrode for 1 mM caffeine under the optimized pH 4 ABS was investigated (Figure 5). Although it is customary that the peak current increases with step potential and pulse amplitude, it is necessary to optimize these parameters by compromising the peak current enhancement with the accompanied peak broadening (capacitive peak current). Taking both the peak current enhancement and peak shape broadening into consideration, 8 and 75 mV were taken as the optimum pulse step potential and differential pulse amplitude, respectively.

**Figure 6:**
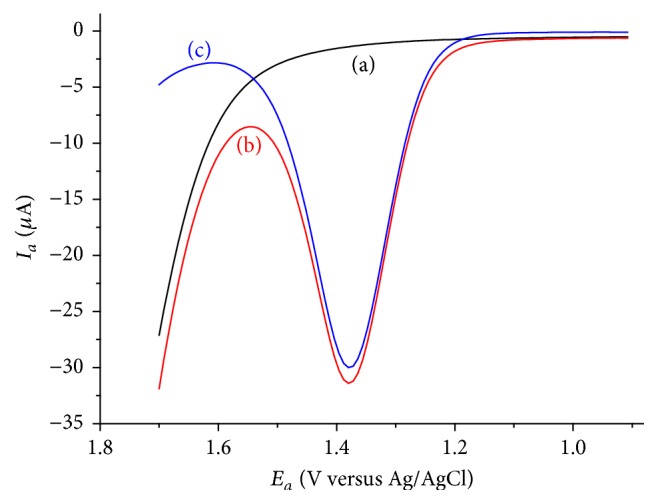
DPVs of LGCE in pH 4 ABS containing (a) no, (b) 1 mM caffeine, and (c) 1 mM caffeine corrected for the blank under optimized conditions.

After optimizing the method and solution parameters, DPV voltammograms were obtained for the LGCE in pH 4.0 ABS containing no (a), 1 mM caffeine (b), and the corrected voltammogram for the blank (c) (Figure 6).

As can be observed from the figure, closeness of the peak current of the subtracted (c) and unsubtracted (b) voltammograms of the LGCE showed low capacitive current of the modified electrode and hence fitness of the electrode for caffeine determination.

### 3.6. Calibration Curve and Method Detection and Quantification Limits

**Figure 7:**
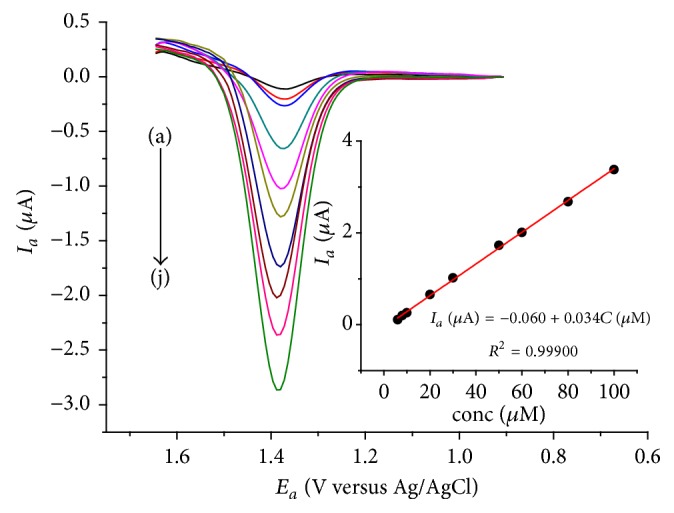
DPVs of LGCE in pH 4.0 ABS containing various concentrations of caffeine ((a)–(j): 6, 8, 10, 20, 30, 40, 50, 60, 80, and 100 *μ*M, resp.). Inset: plot of anodic peak current versus concentration of caffeine.

Under the optimized solution and method parameters, anodic peak current of caffeine at lignin modified GCE was linearly proportional to the caffeine concentration in the range of 6 to 100 × 10^−6^ mol L^−1^ (Figure 7) with a linear regression equation and determination coefficient of *I*_*a*_/*μ*A = −0.060 + 0.034 [Caffeine] *μ*M and *R*^2^ = 0.99900, respectively. Method limit of detection (LoD = 3 s/m) and limit of quantification (LoQ = 10 s/m; where s is blank standard deviation for *n* = 7) were calculated to be 8.37 × 10^−7^ and 2.79 × 10^−6^ M, respectively, making the developed method comparable with the method using Nafion [31] which is expensive electrode modifier.

### 3.7. Application of the Method for the Determination of Caffeine in Coffee Samples

**Figure 8:**
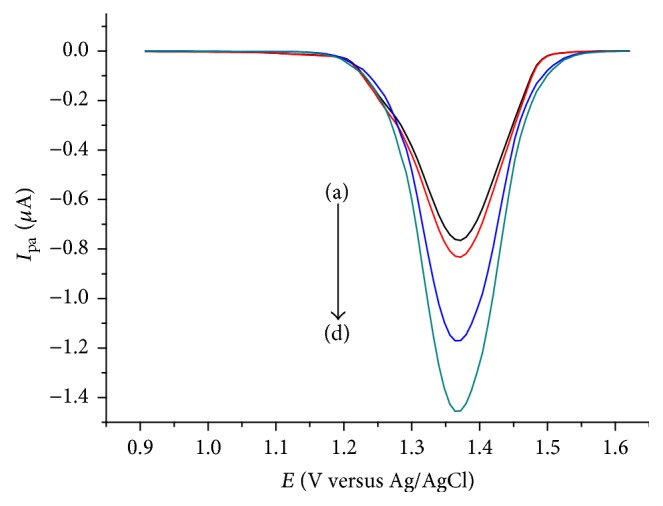
DPVs of LGCE in pH 4 ABS containing aqueous extracts of coffee samples cultivated in different localities of Ethiopia ((a)–(d): Zegie, Finoteselam, Wolga, and Wonbera, resp.).

Aqueous coffee extract samples were prepared as described in the procedure under the experimental part. To determine the concentration of caffeine in real coffee samples cultivated in four different localities of Ethiopia, the differential pulse voltammetric peak current for each extract sample was recorded which then was converted to concentration units using the regression equation of the calibration curve.  Figure 8 presents the DPVs for the coffee extracts of the analyzed four coffee types.  Table 1 presents the summary of the amount of caffeine in the analyzed coffee samples expressed in different units. As can be seen from the table, the caffeine content of the four coffee samples in an increasing order is Zegie, Finoteselam, Wolega, and Wonbera which is in agreement with the reported trend [39, 40]. The observed difference in caffeine content could be attributed to the difference in the agronomical differences between the localities where they were cultivated. This also indicated that the highest and least preference of the people around the localities for Wonbera cultivated coffee and Zegie cultivated coffee, respectively, might be associated with the caffeine content of the coffees.

### 3.8. Recovery Study of the Developed Method

**Figure 9:**
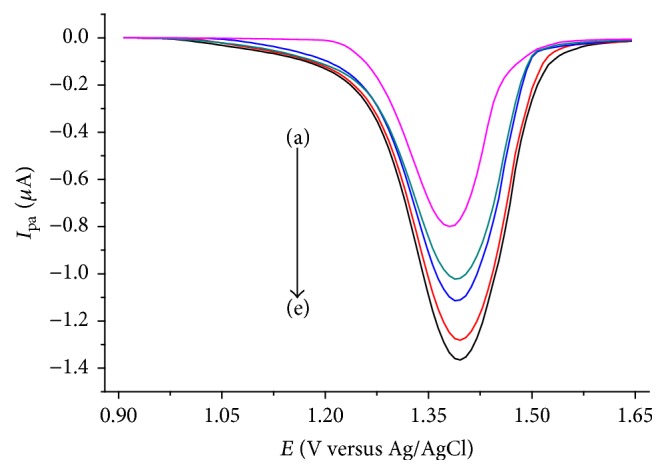
DPVs of aqueous Wolega coffee extract samples spiked with different volumes of 60 *μ*M of standard caffeine. ((a–e): 0, 2, 3, 6, and 7 mL, resp.).

To evaluate the accuracy of the developed DPV method using lignin modified GCE, the recovery of spiked standard caffeine in aqueous extracts of Wolega coffee, which showed an intermediate amount of caffeine among the studied coffee samples, was checked.  Figure 9 presents the voltammograms of an aqueous extracts of Wolega coffee detected to contain 26.31 *μ*M caffeine spiked with different volumes of 60 *μ*M standard caffeine.

As can be seen from the figure, the DPV anodic peak current increased with increasing volume of the spiked standard, indicating the sensitivity of the method developed. Excellent recovery results in the range 93.79–102.17% (Table 2) were obtained, indicating the applicability of the developed method for the determination of caffeine in a complex aqueous coffee extracts.

## 4. Conclusion

This work presents the differential pulse voltammetric determination of water extracted caffeine content of coffee samples cultivated in different localities of Ethiopia using a lignin modified glassy carbon electrode. The electrode modifier reported is relatively cheap, environmentally friendly, easily deposited at the electrode surface exhibiting low capacitive current, and easy to clean.

Differential pulse voltammetric current response showed linear dependence on the concentration in the range 6–100 *μ*M with a linear regression equation, LoD and LoQ of *I*_*a*_/*μ*A = −0.060 + 0.034 [Caffeine] *μ*M, 8.37 × 10^−7^ M, and 2.79 × 10^−6^ M, respectively. Low detection limit, high precision, and excellent recovery results indicated the applicability of the developed method for the electrochemical determination of caffeine in water extracts of real samples of coffee without any interference. Caffeine contents of the coffee samples from four localities (Wonbera, Wolega, Finoteselam, and Zegie) of the origin of coffee, Ethiopia, were found in the order of (mg g^−1^) 10.78, 8.78, 6.35, and 5.85, respectively. From the result, it seems that the highest and lowest preference of the coffee consumers around the study area for Wonbera and Zegie cultivated coffee, respectively, is associated with the caffeine content of the coffee varieties.

## Figures and Tables

**Table 1:** Summary of concentration of caffeine in aqueous coffee extract and corresponding amount of caffeine per gram of roasted coffee of coffee samples from different localities of Ethiopia.

Locality of coffee sample	DPV oxidative peak current (*μ*A)	Caffeine concentration (*μ*A)	Caffeine (mg g^−1^)^*∗*^
Wonbera	1.45	44.41	10.78
Wolega	1.17	36.18	8.78
Finoteselam	0.83	26.18	6.35
Zegie	0.76	24.12	5.85

^*∗*^Caffeine content per gram mass of powdered coffee sample.

**Table 2:** Summary of percentage recoveries of spiked standard caffeine in aqueous coffee extract.

Sample	Initial *μ*M	Spiked standard (*μ*M)	Expected, *μ*M	% recovery
Coffee extract (a)	26.31	—	—	—
Coffee extract (b)	26.31	5.43	5.45	99.63
Coffee extract (c)	26.31	8.00	7.83	102.17
Coffee extract (d)	26.31	12.99	13.85	93.79
Coffee extract (e)	26.31	15.33	15.56	98.52
